# Palmitoylethanolamide Reduces Colon Cancer Cell Proliferation and Migration, Influences Tumor Cell Cycle and Exerts In Vivo Chemopreventive Effects

**DOI:** 10.3390/cancers13081923

**Published:** 2021-04-16

**Authors:** Ester Pagano, Tommaso Venneri, Giuseppe Lucariello, Donatella Cicia, Vincenzo Brancaleone, M. Francesca Nanì, Nunzio A. Cacciola, Raffaele Capasso, Angelo A. Izzo, Francesca Borrelli, Barbara Romano

**Affiliations:** 1 Department of Pharmacy, School of Medicine and Surgery, University of Naples Federico II, 80131 Naples, Italy; ester.pagano@unina.it (E.P.); tommaso.venneri@unina.it (T.V.); giuseppe.lucariello@unina.it (G.L.); donatella.cicia@unina.it (D.C.); mariafrancesca.nani@unina.it (M.F.N.); aaizzo@unina.it (A.A.I.); 2 Department of Science, University of Basilicata, 85100 Potenza, Italy; vincenzo.brancaleone@unibas.it; 3 Research Institute on Terrestrial Ecosystems, National Research Council of Italy (CNR), 80131 Naples, Italy; nunzio.cacciola@iret.cnr.it; 4 Department of Agricultural Sciences, University of Naples Federico II, 80055 Portici, Italy; rafcapas@unina.it

**Keywords:** colon cancer, endocannabinoid system, acylethanolamides

## Abstract

**Simple Summary:**

Treatment of colon cancer remains a significant unmet need. Palmitoylethanolamide (PEA) is an endogenous fatty acid amide also present in food sources. PEA exerts intestinal anti-inflammatory effects, but knowledge of its role in colon carcinogenesis is still largely fragmentary. Here, we found that ultramicronized PEA inhibited tumor cell proliferation mediated by PPAR-α and GPR55, induced cell cycle arrest in the G2/M phase and DNA fragmentation, reduced cell migration and exerted beneficial effects in the azoxymethane model of colonic tumors. Collectively, these data provide evidence on the beneficial effects of PEA in colon carcinogenesis.

**Abstract:**

Palmitoylethanolamide (PEA) is an endogenous fatty acid amide related to the endocannabinoid anandamide. PEA exerts intestinal anti-inflammatory effects, but knowledge of its role in colon carcinogenesis is still largely fragmentary. We deepened this aspect by studying the effects of PEA (ultramicronized PEA, um-PEA) on colon cancer cell proliferation, migration and cell cycle as well as its effects in a murine model of colon cancer. Results showed that um-PEA inhibited tumor cell proliferation via peroxisome proliferator-activated receptor α and G protein-coupled receptor 55, induced cell cycle arrest in the G2/M phase, possibly through cyclin B1/CDK1 upregulation, and induced DNA fragmentation. Furthermore, um-PEA reduced tumor cell migration by reducing MMP2 and TIMP1 expression. In vivo administration of um-PEA exerted beneficial effects in the azoxymethane model of colonic tumors, by reducing the number of preneoplastic lesions and tumors. Collectively, our findings provide novel proofs on the effects of um-PEA in colon carcinogenesis.

## 1. Introduction

Colorectal cancer (CRC) is one of the leading causes of cancer-related death, with a rate of incidence that is remarkably increasing worldwide [1]. It was estimated that 147,950 individuals would be newly diagnosed with CRC in the US in 2020, with 17,930 new cases (12%) in individuals aged younger than 50 years [2]. Similarly, CRC has been placed at the second position in the oncologic death ranking for European countries in 2018 [3]. Risk factors include lifestyle, diet, genetic factors, alterations in gut microbiota, a history of inflammatory bowel disease (IBD) and other additional pathologies (e.g., diabetes, obesity).

Palmitoylethanolamide (PEA) is an endogenous fatty acid amide belonging to the family of acylethanolamides (NAEs), which also includes anandamide (AEA) and oleoylethanolamide (OEA). PEA exerts anti-inflammatory, analgesic and neuroprotective properties with a multitarget mechanism, mostly mediated by peroxisome proliferator-activated receptor α (PPAR-α) [4].

PEA improves inflammation in murine [5,6] and human colon tissues [7], which could be suggestive of a possible role for PEA in intestinal cancer, in light of the well-established association between intestinal inflammation and carcinogenesis [8]. Furthermore, PEA has been shown to: (i) slow down melanoma cell survival in vivo and in vitro [9]; (ii) induce cell death in high-grade astrocytoma/neuroblastoma cells [10]; (iii) be decreased in human brain tumor tissues compared with healthy brain areas [11] and (iv) potentiate the cytotoxic effect of anandamide on human breast cancer cells [12]. Finally, PEA’s targets (e.g., PPAR-α, TRPV1 and CB receptors) are involved in a number of carcinogenesis mechanisms [13]. However, the knowledge on PEA’s effects in colon carcinogenesis is still largely fragmentary, with only one study reporting the antiangiogenic effects of PEA on a human colon carcinoma cell line mediated by VEGF downregulation [14].

Considering the paucity of data on PEA and CRC, this study is aimed at covering this gap by assessing PEA’s effects on colorectal cancer cell proliferation, migration and cell cycle as well as evaluating its possible in vivo effects in a murine model of colon cancer.

## 2. Results

### 2.1. Palmitoylethanolamide Reduces Colon Cancer Cell Proliferation without Affecting the Proliferation of Healthy Colonic Epithelial Cells

**Figure 1:**
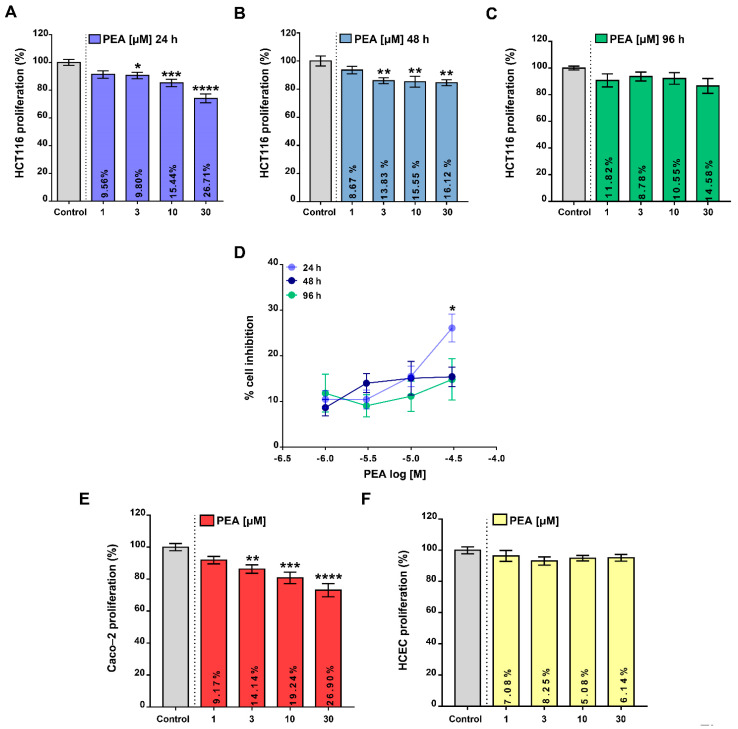
Palmitoylethanolamide affects tumor cell proliferation. Cell proliferation rate of the colorectal cancer cell line HCT116 alone or in the presence of ultramicronized palmitoylethanolamide (PEA, 1–30 μM) for 24 (**A**), 48 (**B**) and 96 h (**C**). In (**D**) the effect of the time course of PEA pharmacological treatment (up to 96 h) is reported, expressed as a percentage of cell proliferation inhibition. * *p* < 0.05 vs. 48 and 96 h as assessed by two-way ANOVA. Effect of PEA treatment (1–30 μM for 24 h) on Caco-2 cells (**E**) and human healthy colonic epithelial cells (HCEC) (**F**). All the data are expressed as a percentage of cell proliferation (n = 3 or 5 independent experiments). Inserted into the columns, the means of the % of cell proliferation inhibition versus the control are reported. All the values are expressed as means ± SEM; * *p* < 0.05, ** *p* < 0.01, *** *p* < 0.001 and **** *p* < 0.0001 vs. control, as assessed by one-way ANOVA followed by Dunnett’s multiple comparisons.

In order to depict the potential role of PEA in intestinal carcinogenesis, we initially tested whether ultamicronized PEA (um-PEA) treatment was able to modify any colorectal cancer cell function in vitro. Firstly, by using the BrdU incorporation assay, we tested the antiproliferative effect of um-PEA on tumor HCT116 cells. Our results revealed that um-PEA (1–30 μM) reduced, in a concentration-dependent manner, the proliferation rate up to 96 h of exposure (Figure 1A–C). Worthy of note, the 30 μM um-PEA concentration was significantly more effective in reducing cell proliferation after 24 h compared with the other timepoints (Figure 1D). Importantly, um-PEA (1–30 µM, 24 h) was also able to significantly reduce the proliferation rate of non-metastatic CRC Caco-2 cells (Figure 1E). Of relevance, um-PEA, at all concentrations tested (1–30 μM, 24 h), did not affect the proliferation rate of the human healthy colonic epithelial cell line (HCEC, Figure 1F). Moreover, um-PEA tested at the highest concentration (30 μM, 24 h) did not change any HCEC cell cycle phases (Figure S1), thus confirming the selective effect of um-PEA in tumoral cells.

### 2.2. Palmitoylethanolamide Reduces CRC Cell Proliferation via PPAR-α and GPR55

**Figure 2:**
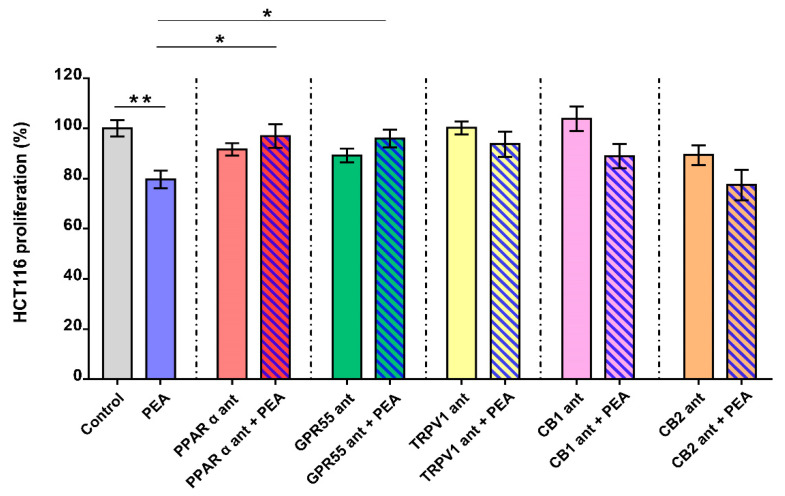
Palmitoylethanolamide’s pharmacological mechanism of action. Antiproliferative effect of the submaximal concentration of ultramicronized palmitoylethanolamide (PEA, 10 μM, 24 h) evaluated in the presence of GW6471 (3 μM, PPARα antagonist, red column); ML181 (10 μM, GPR55 antagonist, green column); 5′-iodoresiniferatoxin (0.1 μM, TRPV1 antagonist, yellow column); AM251 (1 μM, CB_1_ antagonist, pink column) and AM630 (1 μM, CB_2_ antagonist, orange column). All results are expressed as percentage of cell proliferation (*n* = 4 independent experiments) and as means ± SEM; ** *p* < 0.01 vs. control; * *p* < 0.05 vs. PEA as assessed by one-way ANOVA followed by Tukey multiple comparisons test.

Next, we focused on the pharmacological mechanism of action underlying the observed antiproliferative effect of um-PEA on HCT116 cells. We treated such CRC cells with the submaximal concentration of um-PEA (i.e., 10 μM for 24 h) in the presence of selective antagonists of its main targets (i.e., GW6471 (PPAR-α antagonist), 5′-iodoresiniferatoxin (TRPV1 antagonist), ML191 (GPR55 antagonist), AM251 (CB1 antagonist) and AM630 (CB2 antagonist)) [15]. We found that the antiproliferative effect of um-PEA was partially counteracted by GW6471 and ML191, but not by the other antagonists tested (Figure 2). Overall, these results suggest that the antiproliferative effects of um-PEA are, at least in part, mediated by PPAR-α and by GPR55 receptors.

### 2.3. Palmitoylethanolamide Promotes Cell Cycle Arrest in the G2/M Phase in CRC Cells by Promoting DNA Damage

**Figure 3:**
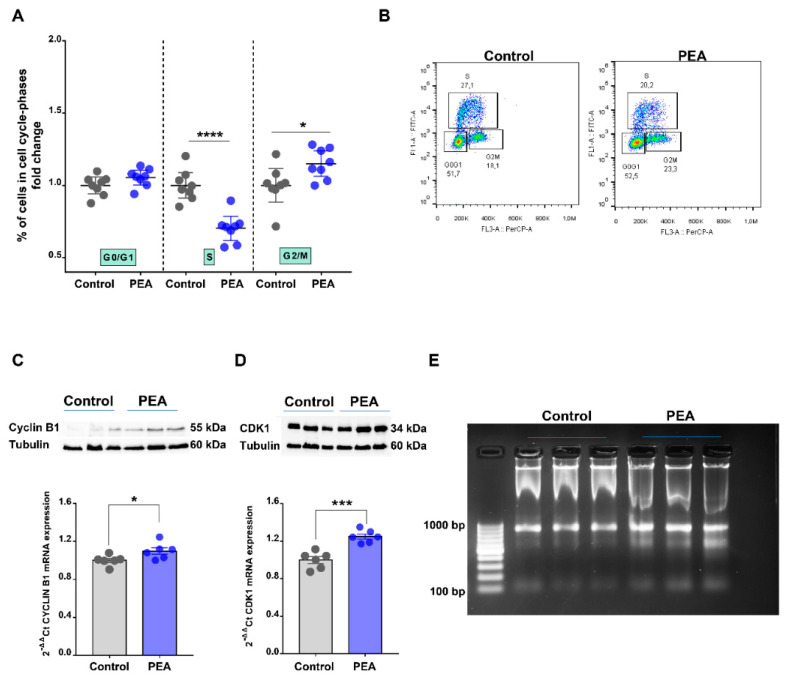
Palmitoylethanolamide induces cell cycle arrest. (**A**) Cell cycle analysis of HCT116 cells treated or not with ultramicronized palmitoylethanolamide (PEA, 30 μM, 24 h) by flow cytometry. Results are expressed as fold change of the cells in each cell cycle phase (*n* = 8). Values are expressed as means ± SEM; * *p* < 0.05 and **** *p* < 0.0001 vs. control (Student’s *t*-test). (**B**) Representative density plots and cell percentage, indicating the G0/G1, S and G2/M phase distribution of HCT116 cells treated or not with um-PEA (30 μM, 24 h). (**C**) Gene and protein expressions of cyclin B1 and (**D**) cyclin-dependent kinase 1 (CDK1) in HCT116 cells, alone or in the presence of PEA (30 μM, 24 h). Gene expression was measured by reverse transcription quantitative polymerase chain reaction (RT-qPCR) and calculated by using the 2-ΔΔCt formula (*n* = 6), whereas protein expression was evaluated by Western blot analysis and normalized to the housekeeping protein α-tubulin (*n* = 3). Values are expressed as means ± SEM; * *p* < 0.05 and *** *p* < 0.001 vs. control as assessed by Student’s *t*-test. (**E**) Detection of DNA ladder in HCT116 treated (um-PEA) or not (ctrl) with um-PEA (30 μM, 24 h) by standard agarose gel electrophoresis. The first lane represents the standard molecular size marker. Detailed information about Western Blot can be found at Supplementary Materials.

In an attempt to further deepen our understanding of um-PEA’s effects on HCT116 cells, we explored its ability to affect HCT116 cell cycle progression by using BrdU/7-AAD staining. Flow cytometry analysis revealed that um-PEA treatment (30 μM for 24 h) led to an accumulation of cells harboring the G2/M phase (Figure 3A, see also the representative density plots reported in Figure 3B) with a concomitant significant reduction in the proportion of cells in the S phase. It is well-established that G2/M transition is regulated by the cyclin B1/CDK1 complex [16]. Thus, we investigated the potential role of this complex in the um-PEA action. Our study revealed that um-PEA treatment resulted in an increased expression (both mRNA and protein) of the cyclin B1/CDK1 complex (Figure 3C,D), thus suggesting that um-PEA inducing G2/M phase arrest may be related to sustained activation of the main executors of the G2/M phase.

We also investigated the involvement of the mTOR signaling pathway, since it is well-known for its role in regulating tumor cell growth and its implication in cancer [17]. Thus, we investigated the expression of the main actors of this pathway, namely phospho-p44/42 MAP kinase (Erk1/2), phospho S6 ribosomal protein, phospho AKT and phospho proline-rich Akt substrate (pPRAS40). Results showed no differences between control and um-PEA treatment (Figure S2). Moreover, in order to verify whether um-PEA induced DNA damage of CRC cells, consequently inducing G2/M arrest, we performed the DNA fragmentation assay. Um-PEA treatment (30 μM for 24 h) determined the formation of a clearly distinguishable DNA ladder in gel electrophoresis (Figure 3E), thus confirming the functional relationship between the cell cycle G2/M arrest and the induction of DNA damage upon um-PEA treatment.

### 2.4. Palmitoylethanolamide Reduces Tumor Cell Migration

**Figure 4:**
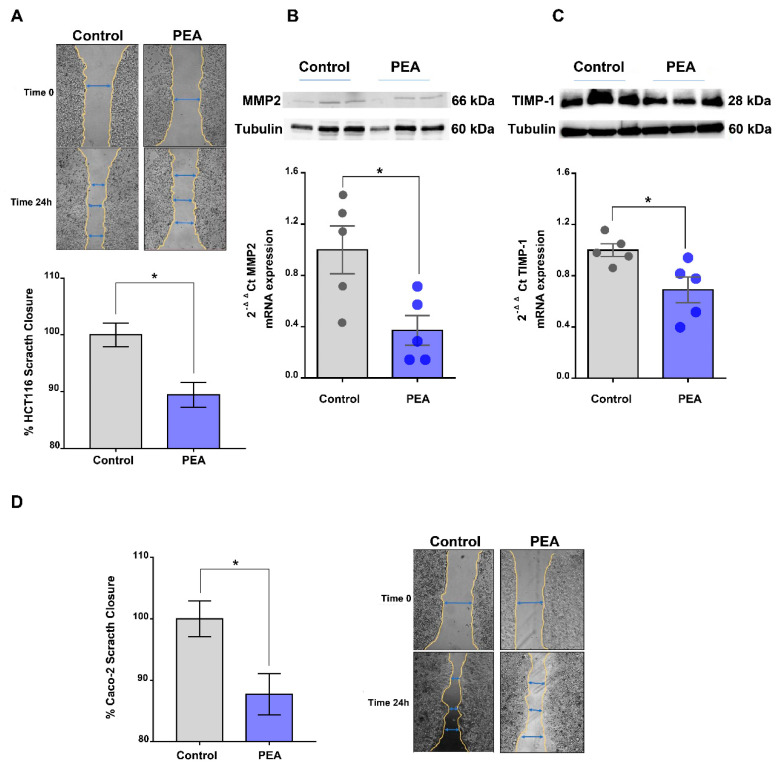
Palmitoylethanolamide affects tumor cell migration. (**A**) In vitro scratch assay performed in HCT116 cells alone or in the presence of ultramicronized palmitoylethanolamide (PEA, 30 μM, 24 h). The scratches are expressed as a percentage of scratch closure (*n* = 4 experiments). Representative images of the scratch areas, at time zero and 24 h, of HCT116 cells alone or in the presence of PEA (30 μM, 24 h) are reported on top of the graph. Gene and protein expressions of matrix metalloproteinase type 2 (MMP2). Magnification: 4×. (**B**) and tissue inhibitor matrix metalloproteinase 1 (TIMP1) (**C**) in HCT116 cells, alone or in the presence of PEA (30 μM, 24 h). Gene expression was measured by RT-qPCR and calculated by using the 2-ΔΔCt formula (*n* = 5), whereas protein expression was evaluated by Western blot analysis and normalized to the housekeeping protein α-tubulin (*n* = 3). Detailed information about Western Blot can be found at Supplementary Materials. (**D**) In vitro scratch assay performed in Caco-2 cells alone or in the presence of PEA (30 μM, 24 h). The scratches are expressed as percentage of scratch closure (*n* = 4 experiments). Next to the graph, representative images of the scratch areas at time zero and time 24 h for Caco-2 cells alone or in the presence of PEA (30 μM, 24 h) are reported. Values are expressed as means ± SEM; * *p* < 0.05 vs. control as assessed by Student’s *t*-test. Magnification: 4×.

Tumor cell migration is essential for invasion and dissemination from primary solid tumors and for the spread of the tumor cells [18]. Scratch assay analysis, a well-known method to study cell migration, revealed that um-PEA significantly decreased the migration of HCT116 cells (Figure 4A), when their proliferation was blocked by pretreatment with mitomycin C.

Matrix metalloproteinases (MMPs) are critical molecules implicated in different hallmarks of carcinogenesis including tumor growth and migration [19]. Of relevance, among the MMPs involved in cell migration, we noticed that um-PEA treatment determined a decrease in MMP2 expression (both mRNA and protein) (Figure 4B) without perturbing the expression of other relevant genes involved in epithelial mesenchymal transition (EMT, i.e., one of the main phenomena responsible for epithelial cell invasion and migration involved in cancer progression [20]), namely snail family zinc finger 1 (SNAI1), zinc finger protein SNAI2 (SLUG), twist family BHLH transcription factor 1 (TWIST), transforming growth factor beta (TGF-β), E-cadherin and vimentin (Figure S3A–F). Um-PEA treatment also induced downregulation of mRNA and protein of tissue inhibitor matrix metalloproteinase 1 (TIMP1) (Figure 4C), a key molecule in regulating tumor invasion and proliferation [20]. Also of relevance, um-PEA was able to reduce the migration of another colorectal cancer cell line (i.e., Caco-2) (Figure 4D). Collectively, our data show the role of um-PEA in reducing tumor cell migration as well as tumor proliferation.

### 2.5. Palmitoylethanolamide Prevents Tumor Development in a Murine Model of Colon Cancer

**Figure 5:**
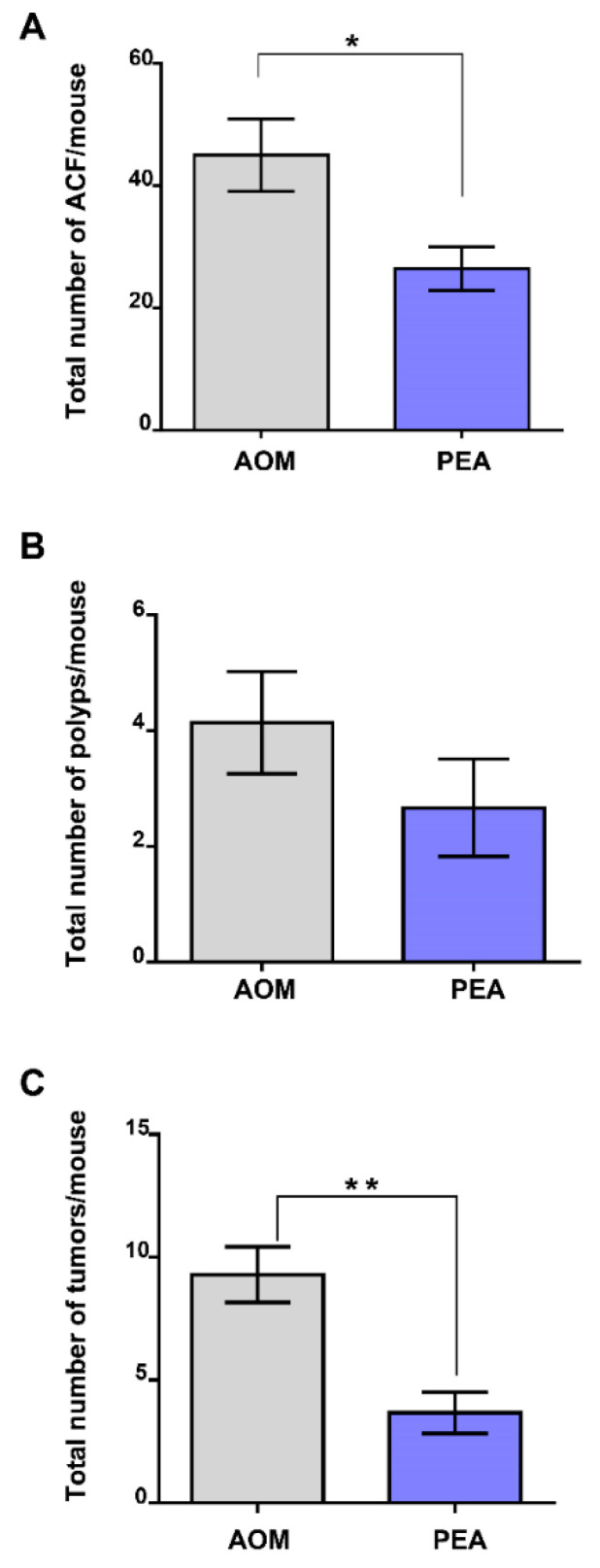
Effect of palmitoylethanolamide treatment in the murine azoxymethane (AOM) model of colon cancer. Total number of aberrant crypt foci (ACF) (**A**), polyps (**B**) and tumors (**C**) derived from the administration of AOM (40 mg/kg, intraperitoneally) in mice treated or not with ultramicronized palmitoylethanolamide (PEA, 10 mg/kg, intraperitoneally, three times/week for 13 weeks), (*n* = 6–7 mice per experimental group). Results are expressed as means ± SEM; * *p* < 0.05 and ** *p* < 0.01 vs. AOM as assessed by Student’s *t*-test.

In order to shed light on the effects of um-PEA in vivo, we took advantage of the azoxymethane (AOM) model of colon cancer, a useful murine model to study CRC development and progression [21]. The carcinogenic agent AOM, given alone, induced the likely presence of aberrant crypt foci (ACF) (Figure 5A), polyps (Figure 5B) and tumors (Figure 5C). Our data revealed that um-PEA (10 mg/kg), injected intraperitoneally every two days for 13 weeks, significantly reduced the number of AOM-induced ACF preneoplastic lesions (Figure 5A) as well as the total number of tumors (Figure 5C). Although not statistically significant, um-PEA exhibited a trend in reducing also the number of polyps (35.63% inhibition) (Figure 5B). In light of our data, we conclude that um-PEA administration is effective in reducing colorectal cancer development, thus showing chemopreventive effects.

## 3. Discussion

Taking into account the epidemiological data reporting a high rate of deaths related to colorectal cancer (CRC), there is a clinical need for new pharmacotherapeutic options. While PEA’s role in other cancers (e.g., melanoma, breast cancer, neuroblastoma) has been previously documented [9,10,12], its role in CRC is still at a very early stage of understanding [14]. In this study, by investigating um-PEA’s effects on cell proliferation, cell cycle and migration of CRC cell lines as well as in a murine model of colon cancer, we have provided key proofs of the beneficial effects of PEA in colon carcinogenesis. In preliminary experiments, um-PEA’s effect on cell proliferation was evaluated during a time period of 96 h incubation. Because the antiproliferative effect, at least for the 30 µM concentration, was maximal after 24 h of treatment, we selected this timepoint for further evaluations. The gradual disappearance of um-PEA’s effect after 24 h incubation could be due to its enzymatic catabolism [22], since NAAA (i.e., the main PEA hydrolytic enzyme) is expressed in HCT116 cells. Also, the possibility that HCT116 cells could activate biological pathways able to overcome um-PEA’s antiproliferative activity cannot be ruled out.

Here, we have shown that um-PEA exerted antiproliferative effects in two different colon adenocarcinoma cell lines. The antiproliferative effect of PEA on Caco-2 cells has been previously documented [14], with a maximal effect higher than that found in our experiments. Such a discrepancy, which might be due to several reasons, requires further investigations. Nevertheless, our results are in agreement with those reporting the antiproliferative effects of PEA on different tumor cell lines such as melanoma, neuroblastoma and breast cancer cells [9,12,23]. Also of relevance, discrepancies in the potency and efficacy of PEA have been previously documented [24]. Of relevance, um-PEA altered neither the growth of healthy cells nor their cell cycle phases, indicating that the effect of um-PEA is tumor-specific.

In order to depict the mechanism of action behind um-PEA’s antiproliferative effects on tumor cells, we conducted pharmacological studies, in which selective antagonists of PEA’s main targets [15] were combined with um-PEA. Indeed, it has been demonstrated that PEA directly or not activates PPAR-α, GPR55 [4], transient receptor potential cation channel subfamily V member 1 (TRPV1) and cannabinoid (CB) receptors [4]. Here, our data highlight that the antiproliferative effect of PEA in the CRC cells was counteracted by PPAR-α and GPR55 antagonists, suggesting that um-PEA’s antiproliferative behavior is mediated by such targets.

To further explore the effect of PEA on tumor cells, we investigated whether this amide was able to regulate cell cycle progression. We showed the first proof that um-PEA led to colon cancer cell cycle arrest in the G2/M phase, with a parallel decrease in the percentage of cells in the S phase. The G2/M transition is regulated by the cyclin B1/CDK1 complex, a required checkpoint for cell cycle arrest in this phase [16]. We also found that um-PEA induced an increase in the expression of the cyclin B1/CDK1 complex (a required checkpoint for cell cycle arrest in the G2/M transition), which participates, at least in part, in cell cycle arrest in the G2/M phase. Among the other NAEs, only anandamide has been reported to affect tumor cell cycle [25], to date. In order to further depict um-PEA’s mode of action, we also investigated the involvement of the mTOR signaling pathway, which is known to regulate tumor cell growth [17]. In light of our data, in which we did not find any change in the expression of the key actors of the mTOR pathway upon um-PEA treatment, we exclude that this pathway is involved in um-PEA’s mode of action. Although our data are not in line with those reported by Sarnelli et al. on Caco-2 cells [14], they are in agreement with literature data showing that the downstream pathways associated with the activation of PPAR-α and GPR55 (which are the main PEA targets involved in our study) in colorectal cancer are mTOR pathway-independent. Since the G2/M checkpoint is known to be arrested in response to DNA damage [26], we also verified this feature. Our results highlight that um-PEA damages DNA, thus supporting the functional relationship between the cell cycle G2/M arrest and PEA-induced DNA damage.

It will be a specific aim of further investigations to study whether or not PEA affects cancer stemness, a well-known hallmark of cancer progression and growth.

In favor of gaining knowledge on PEA’s effects on colorectal cancer cells, we also investigated the ability of PEA to influence CRC cell migration. We found that um-PEA treatment decreased the migration rate of two CRC cell lines by diminishing the expression of MMP2, a member of the matrix metalloproteinases family, which is known to be implicated in different hallmarks of carcinogenesis (e.g., tumor growth and migration) [19]. Obviously, further insights into MMP2 involvement would arise from experiments in which MMP2 is pharmacologically inhibited. Also of importance, um-PEA reduced the expression of tissue inhibitor matrix metalloproteinase 1 (TIMP1), a pivotal player in regulating the balance of matrix remodeling and in cell proliferation [20], whose suppression is implicated in the decreased progression of colon cancer [24]. Overall, our data support the hypothesis that um-PEA is implicated in cell migration and proliferation, possibly via downregulation of MMP2 and TIMP1.

Finally, we demonstrated that um-PEA exerted beneficial effects in a murine model of colon cancer induced by the administration of AOM. This model has been extensively used to study the mechanisms underlying human sporadic colon cancer as well as to evaluate drugs with potential chemopreventive effects [25]. Importantly, although we performed a quantitative analysis only, without the support of immunohistochemistry, our data clearly demonstrate that um-PEA showed a chemopreventive effect, being able to significantly reduce ACF and tumor number formation and by showing a trend in reducing the number of polyps. ACF, as well as polyps, are well-known precursors of colon cancer in humans [27]. The chemopreventive effects of um-PEA could be related to its ability to attenuate in vivo colonic inflammation [5,6], a well-known risk factor for the development of colon cancer [8].

## 4. Materials and Methods

### 4.1. Drugs

Ultramicronized PEA (um-PEA, powder particle size <10 µm, with the following distribution: <6 µm, 99.9%; <2 µm, 59.6%; <1 µm, 14.7%; <0.6 µm, 2%, as described in patent EP2475352 A1, with text from patent WO2011027373A1) was kindly provided by Epitech Group (Saccolongo, Italy). Azoxymethane (AOM) was obtained from Sigma-Aldrich (Italy). All reagents for cell cultures were purchased from Sigma-Aldrich (Milan, Italy), Bio-Rad Laboratories (Milan, Italy) and Aurogene Srl (Rome, Italy). The vehicles used for in vivo (ethanol/Tween20/saline in a ratio of 1:1:8, 2 mL/kg) and in vitro (0.1% ethanol) experiments had no effects on the responses under study.

### 4.2. Cell Lines

The human colon adenocarcinoma cell lines (i.e., HCT116 and Caco-2) (ATCC from LGC Standards, Milan, Italy) and the immortalized healthy human colonic epithelial cells (HCEC), derived from human colon biopsies, kindly gifted by Fondazione Callerio Onlus (Trieste, Italy) were cultured in Dulbecco’s modified Eagle’s medium (DMEM) (Sigma-Aldrich, Milan, Italy) supplemented with 10% heat-inactivated FBS (Sigma-Aldrich, Milan, Italy). Cell lines were maintained at 37 °C in a humidified incubator with 5% CO_2_, and their viability was evaluated by trypan blue exclusion.

### 4.3. BrdU Incorporation

HCT 116, Caco-2 and HCEC were seeded in 96-well plates (1.0 × 10^4^ cells per well), allowed to adhere (within 24 h) and starved by serum deprivation for 18 h. Tumoral and no-tumoral cells were all treated with um-PEA (1–30 μM). After 24, 48 and/or 72 h of treatment, pulsing cells were incubated with BrdU (10 µM) in the cell medium for 2 h. Thereafter, the proliferation of the cells was determined by using the BrdU proliferation ELISA kit (Roche, Milan, Italy) according to the manufacturer’s instructions. Using this assay, the antiproliferative effect of um-PEA (used at the submaximal concentration 10 μM) was also evaluated (in HCT116 cells) in the presence of GW6471 (3 μM, PPARα antagonist); ML191 (1 μM, GPR55 antagonist); 5′-iodoresiniferatoxin (0.1 μM, TRPV1 antagonist); AM251 and AM630 (both 1 μM, CB1 and CB2 antagonist, respectively) [15] [all from Tocris, Rodano, Italy and incubated 30 min before um-PEA (10 μM)]. All results are expressed as a percentage of cell proliferation (*n* = 4 experiments including 4 replicates for each treatment).

### 4.4. Scratch Assay

Sub-confluent HCT116 and Caco-2 cell lines were trypsinized and plated on a 2-well culture-insert (ibidi GmbH, Gräfelfing, Germany) inserted on a 24-well plate (5 × 10^4^ cells/70 µL) and left to adhere overnight. After this time, the insert was removed, and cells were washed with phosphate buffer saline (PBS 1×) and treated with mitomycin C 30 µg/mL (Sigma-Aldrich, Milan, Italy) in serum-free media, in order to inhibit cell proliferation completely. After 2 h, tumoral cells were treated with um-PEA (30 µM) for 24 h. Wound area recovery was observed under a phase-contrast microscope (Leica, Wetzlar, Germany) and photographed at the time zero point (right after the mitomycin C removal) and after 24 h of treatment. Successively, by using the ImageJ software, the size of the opened area was measured from the digital images. The results are expressed as % of scratch closure (time zero/time 24 h × 100). Two images were acquired for each well, and at least 3 replicates were analyzed for each treatment. Four independent experiments were independently carried out.

### 4.5. Cell Cycle Analysis

Cell cycle analysis was performed according to BD Pharmingen™ BrdU Flow Kit (BD Biosciences, San Jose, CA USA) and conducted on HCT116 cells (1.5 × 10^5^ cells seeded in a 6-well plate), overnight serum deprived and treated or not with um-PEA (30 μM) for 24 h. Cells were revealed by using BriCyte flow cytometer (Mindray, Trezzano sul Naviglio Italy), gated based on forward and side scatter to separate debris, and then the cellular events were further gated based on their BrdU and 7-AAD content. Data were analyzed by FlowJo v10 software (Tree Star, Ashland, OR, USA) and expressed as fold change of the cells in each cell cycle phase.

### 4.6. Gene Expression Analysis by Reverse Transcription Quantitative Polymerase Chain Reaction (RT-qPCR)

mRNA obtained from human cell lines was extracted by using Purezol Reagent (Bio-Rad, Milan, Italy), following the manufacturer’s instructions. Reverse transcription was performed by using the High-Capacity cDNA Reverse Transcription Kit (Applied Biosystems, Foster City, CA, USA), and qPCR was completed using SYBR Green Master Mix (Applied Biosystems, Foster City, CA, USA) and gene specific primers, as detailed in Table 1. All the data were normalized to the housekeeping gene glyceraldehyde-3-phosphate dehydrogenase (GAPDH), and the relative abundance was expressed by using the 2-ΔCt formula.

### 4.7. Western Blot Analysis

Western blot analysis was performed to investigate the expression of cyclin B1, CDK1, MMP2 and TIMP1 in the HCT116 tumoral cell line alone or in the presence of um-PEA (30 μM, 24 h). Cells were lysates in RIPA buffer (10 mM Tris-Cl (pH 8.0), 1 mM EDTA, 140 mM NaCl, 1% Triton X-100, 0.1% (*v*/*v*) sodium deoxycholate, 0.1% SDS) supplemented with protease (Roche, Monza, Italy) and phosphatase inhibitors (Sigma-Aldrich, Milan, Italy). Forty micrograms of protein extract was fractionated by 12% SDS-PAGE according to the manufacturer’s protocols (Bio-Rad, Milan, Italy). After incubation with 5% (*w*/*v*) non-fat milk in TBS-T (10 mM Tris, pH 8.0, 150 mM NaCl, 0.5% (*v*/*v*) Tween-20) for 60 min, the membranes were incubated overnight with anti-Cyclin B1 (1:2000, Cell Signaling, Danvers, MA, USA), anti-CDK1 (1:1000, Invitrogen, Carlsbad, CA, USA), anti-MMP2 (1:1000, Invitrogen, Carlsbad, CA, USA) and anti-TIMP1 (1:200, Invitrogen, Carlsbad, CA, USA), and thereafter, anti-mouse IgG secondary antibodies (1:3000, Cell Signaling, Danvers, MA, USA), linked to horseradish peroxidase, were added. The signal was visualized by enhanced chemiluminescence using Chemidoc XRS (Biorad, Milan, Italy) and analyzed using Image Lab version 6.10.7. α-tubulin (1:1000, Cell Signaling, Danvers, MA, USA) was used as housekeeping normalizing protein.

### 4.8. DNA Fragmentation Assay

HCT116 cells were seeded in 10 cm culture dishes (5 × 10^5^ cells per well), allowed to adhere (within 24 h) and starved for 18 h. Then, cells were treated with vehicle or um-PEA (30 µM). After 24 h of treatment, cells were washed twice in PBS, and incubated in DNA-lysis Buffer (50 mM Tris pH 7.5, 100 mM NaCl, 5 mM ethylenediaminetetraacetic acid, 1% sodium dodecyl sulphate, 0.5 mg/mL proteinase K) for 1 h at 55 °C before extraction with phenol/chloroform/isoamyl alcohol. The suspension was then centrifuged (4000 rpm) for 5 min. DNA was precipitated with 1 volume of 5 M NaCl and 2.5 volumes of 95% (*v*/*v*) ethanol. The isolated DNA was resolved on a 1.5% agarose gel containing GreenSafe DNA Gel Stain (Canvax, Cordoba, Spain) in 40 mM Tris-acetate-EDTA buffer with electrophoresis at 80 V for 30 min. DNA fragments were visualized and photographed under ultraviolet light using Chemidoc XRS (Biorad, Milan, Italy).

### 4.9. Mice

Six-week-old male CD1 background mice were purchased from Charler River (Sant’Angelo Lodigiano, Italy), fed ad libitum with standard food (Mucedola srl, Settimo Milanese, Italy) and housed in polycarbonate cages under a 12 h light/12 h dark cycle at the Department of Pharmacy, University of Naples Federico II. All mice were used after a 1-week acclimation period (temperature 23 ± 2 °C; humidity 60%, free access to water and food). All the experimental procedures and protocols were in conformity with the principles of laboratory animal care, in compliance with national (Direttiva 2010/63/UE) laws and policies and approved by the Italian Ministry of Health. All studies involving animals are reported in accordance with the ARRIVE guidelines for reporting experiments involving animals [28].

### 4.10. Azoxymethane (AOM) Murine Model of Colon Cancer

The effect of um-PEA was evaluated in a murine model of chemically AOM-induced colon cancer. AOM (40 mg/kg in total, intraperitoneally (ip) was administered in mice at the single dose of 10 mg/kg once per week for four weeks. Um-PEA at a dose of 10 mg/kg was given (ip) three times per week for all the duration of the experiment, starting one week before the first administration of AOM in order to appreciate its chemopreventive effect. Um-PEA dose was selected on the basis of previous published work which showed the in vivo selective pharmacological effect of PEA in the intestine [5,29,30]. All mice were humanely euthanized 12 weeks after the first injection of AOM. Based on our laboratory experience, this time (at the used dose of AOM) was associated with the occurrence of a significant number of aberrant crypt foci (ACF, which are considered preneoplastic lesions), polyps and tumors [31]. Detection and quantization of ACF, polyps and tumors on the colon were performed as previously reported [32].

### 4.11. Statistical Analysis

Data are expressed as mean ± SEM of n experiments. To determine statistical significance, Student’s *t*-test was used for comparing a single treatment mean with a control mean, and a one-way ANOVA followed by a Tukey multiple comparisons test and/or Dunnett’s multiple comparisons test was used for the comparison of multiple groups. Two-way ANOVA was used to compare different concentration–effect curves. A *p*-value < 0.05 was considered to be significant. G Power was used for sample size calculation.

## 5. Conclusions

In this study, we provide evidence for the beneficial effects of PEA in colon carcinogenesis. Um-PEA pharmacological treatment inhibited tumor cell proliferation in vitro with a mechanism likely mediated by PPAR-α and GPR55. Notably, um-PEA induced cell cycle arrest in the G2/M phase (possibly through cyclin B1/CDK1 upregulation and inducing DNA damage) and reduced tumor cell migration by downregulating MMP2 and TIMP1 expression. In vivo, um-PEA exerted chemopreventive effects by reducing the number of preneoplastic lesions (i.e., ACF) and tumors.

## Figures and Tables

**Table 1:** Sequences for *Homo sapiens* primers, representing the genes screened by quantitative polymerase chain reaction (qPCR).

Targeted Gene	Forward Sequence	Reverse Sequence
*CDK1*	GGATGTGCTTATGCAGGATTCC	CATGTACTGACCAGGAGGGATAG
*CYCLIN B1*	AACTTTCGCCTGAGCCTATTTT	TTGGTCTGACTGCTTGCTCTT
*E-CADHERIN*	ATTTTTCCCTCGACACCCGAT	TCCCAGGCGTAGACCAAGA
*GAPDH*	GGAGCGAGATCCCTCCAAAAT	GGCTGTTGTCATACTTCTCATGG
*MMP2*	CCCACTGCGGTTTTCTCGAAT	CAAAGGGGTATCCATCGCCAT
*SLUG*	TGTGACAAGGAATATGTGAGCC	TGAGCCCTCAGATTTGACCTG
*SNAI1*	ACTGCAACAAGGAATACCTCAG	GCACTGGTACTTCTTGACATCTG
*TGF-β*	CTAATGGTGGAAACCCACAACG	TATCGCCAGGAATTGTTGCTG
*TIMP1*	CTTCTGCAATTCCGACCTCGT	ACGCTGGTATAAGGTGGTCTG
*TWIST*	GTCCGCAGTCTTACGAGGAG	GCTTGAGGGTCTGAATCTTGCT
*VIMENTIN*	AGTCCACTGAGTACCGGAGAC	CATTTCACGCATCTGGCGTTC

## Data Availability

The data presented in this study are available on request from the corresponding author.

## Supplementary Materials

The following are available online at https://www.mdpi.com/article/10.3390/cancers13081923/s1, Figure S1: The effect of palmitoylethanolamide on cell cycle of healthy colonic epithelial cells, Figure S2: mTOR signaling pathway upon palmitoylethanolamide treatment, Figure S3: Palmitoylethanolamide effects on epithelial to mesenchymal transition (EMT). Original Western Blot figures.
