# Supplementary material for: Palmitoylethanolamide Reduces Colon Cancer Cell Proliferation and Migration, Influences Tumor Cell Cycle and Exerts In Vivo Chemopreventive Effects
Source: Cancers (Basel). 2021 Apr 16;13(8):1923. doi: 10.3390/cancers13081923 (PMC8073478; doi:10.3390/cancers13081923)
Supplement: Supplementary file 1 [file cancers-13-01923-s001.zip › cancers-1181723-supplementary.pdf]

*Supplementary Materials*

# Palmitoylethanolamide Reduces Colon Cancer Cell Proliferation and Migration, Influences Tumor Cell Cycle and Exerts In Vivo Chemopreventive Effects

**Ester Pagano, Tommaso Venneri, Giuseppe Lucariello, Donatella Cicia, Vincenzo Brancaleone, M. Francesca Nanì, Nunzio A. Cacciola, Raffaele Capasso, Angelo A. Izzo, Francesca Borrelli and Barbara Romano**

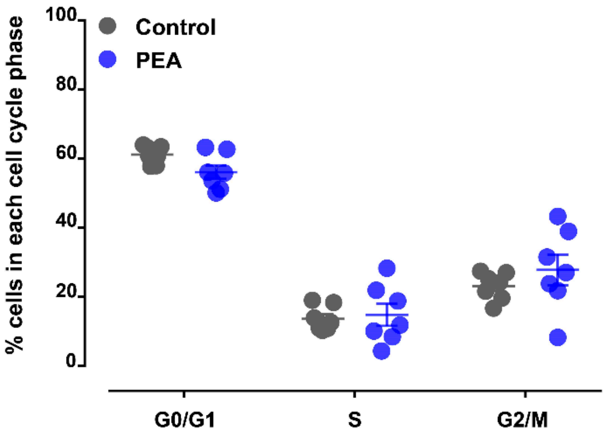


**Figure S1.** The effect of palmitoylethanolamide on cell cycle of healthy colonic epithelial cells. Cell cycle analysis of human healthy colonic epithelial cells (HCEC) treated or not with ultramicronized palmitoylethanolamide (PEA, 30 µM, 24 h) by flow cytometry. Results are expressed as percentage of cells in each cell cycle-phases ($n$ = 7). Values are expressed as means ± SEM and Student's $t$-test was performed.

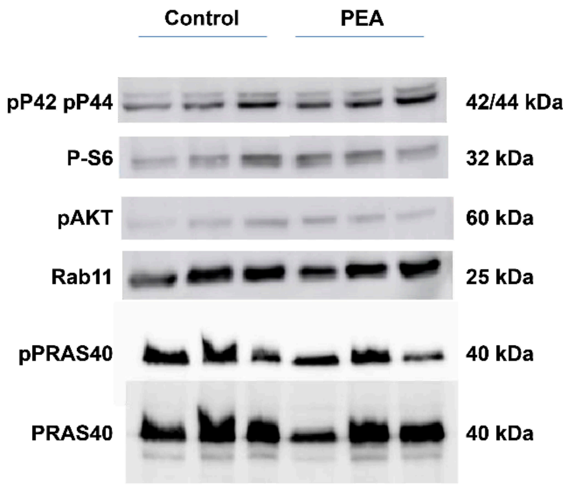


**Figure S2.** mTOR signaling pathway upon palmitoylethanolamide treatment. Protein expressions, in HCT116 cells alone on in presence of ultramicronized palmitoylethanolamide (PEA, 30 µM, 24 h), of phospho-p44/42 MAP kinase (Erk1/2) (first band), phospho S6 ribosomal protein (second band), phospho AKT (third band) normalized on the housekeeping protein Ras-related protein (Rab11, fourth band) as well as of phospho proline-rich Akt substrate (pPRAS40, fifth band) normalized on its no-phosporilated form (PRAS40, sixth band) ($n$ = 3).

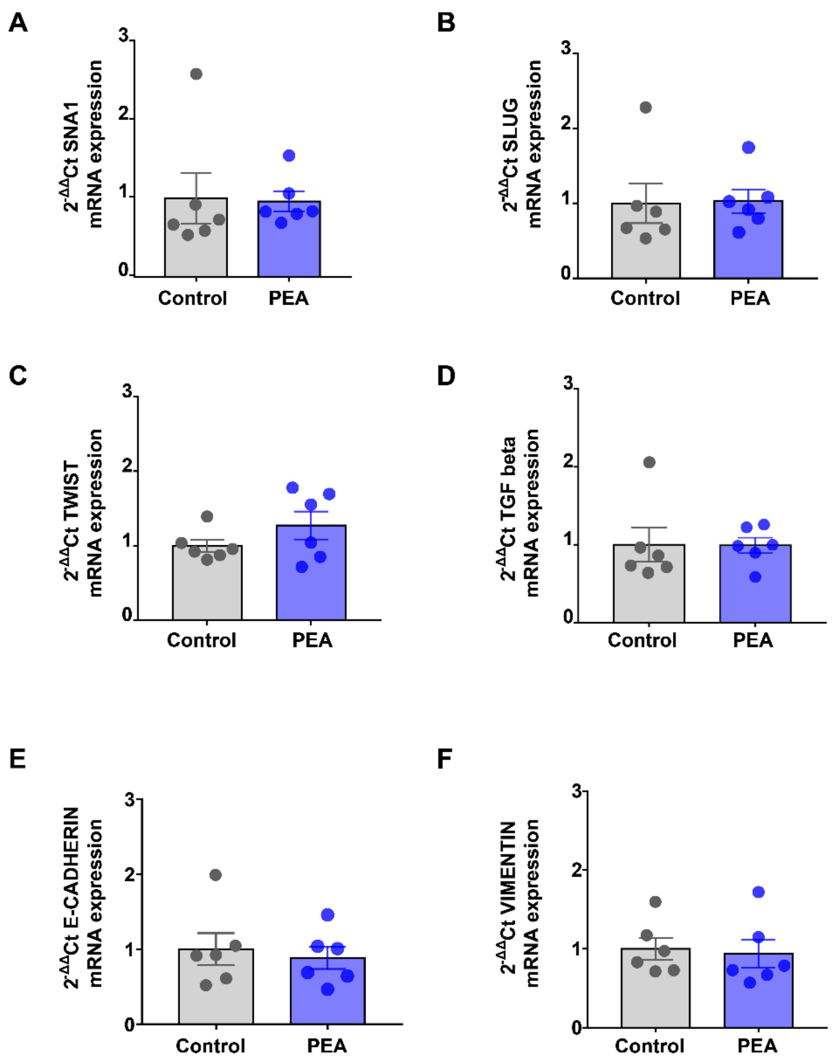


**Figure S3.** Palmitoylethanolamide effects on epithelial to mesenchymal transition (EMT). Gene expressions of SNAI1 (Snail Family Zinc Finger 1, **A**), SLUG (Zinc Finger Protein SNAI2, **B**), TWIST (twist family BHLH transcription factor 1, **C**), TGF-β (transforming growth factor beta, **D**), E-cadherin (**E**) and Vimentin (**F**) in HCT116 cells, alone or in presence of ultramicronized palmitoylethanolamide (PEA, 30 μM, 24 h). Gene expression was measured by qRT-PCR and calculated by using the 2-ΔΔCt formula (*n* = 6). Values are expressed as means ± SEM and Student's *t*-test was performed.

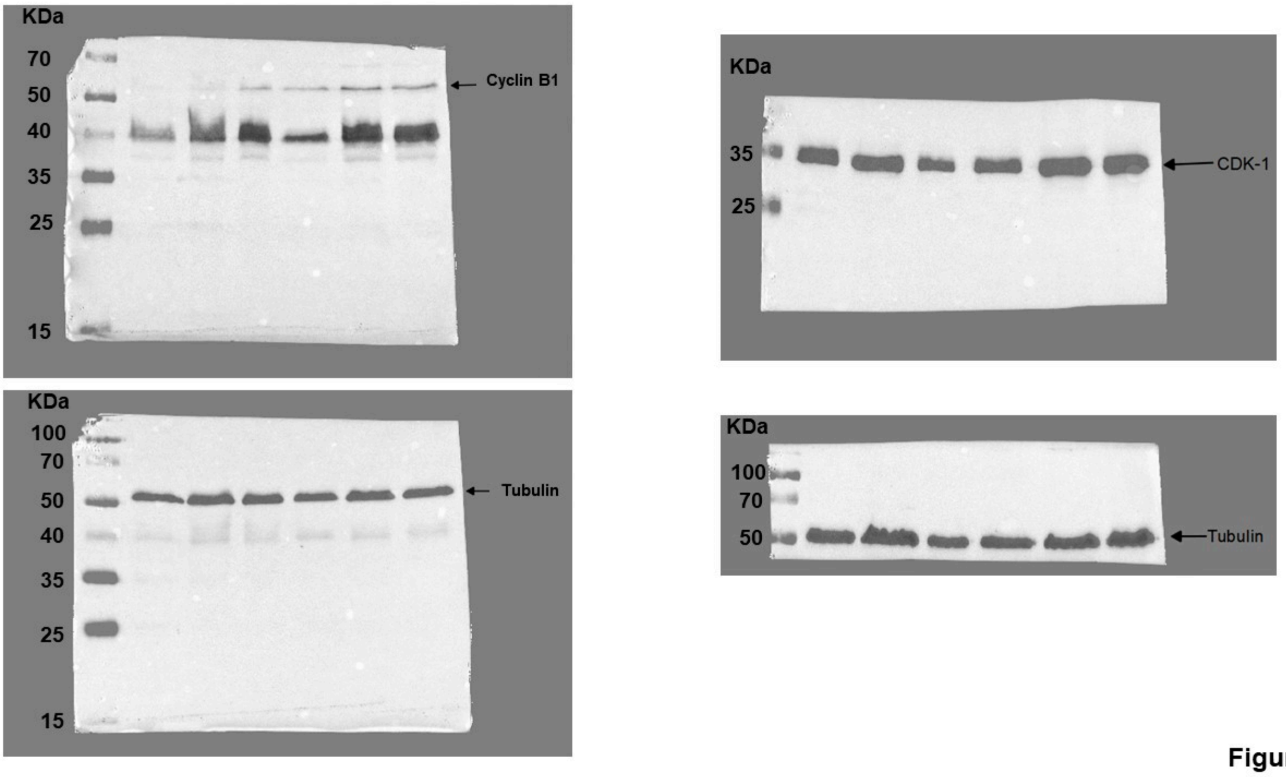


Figure 3

Detailed information about Figure 3.

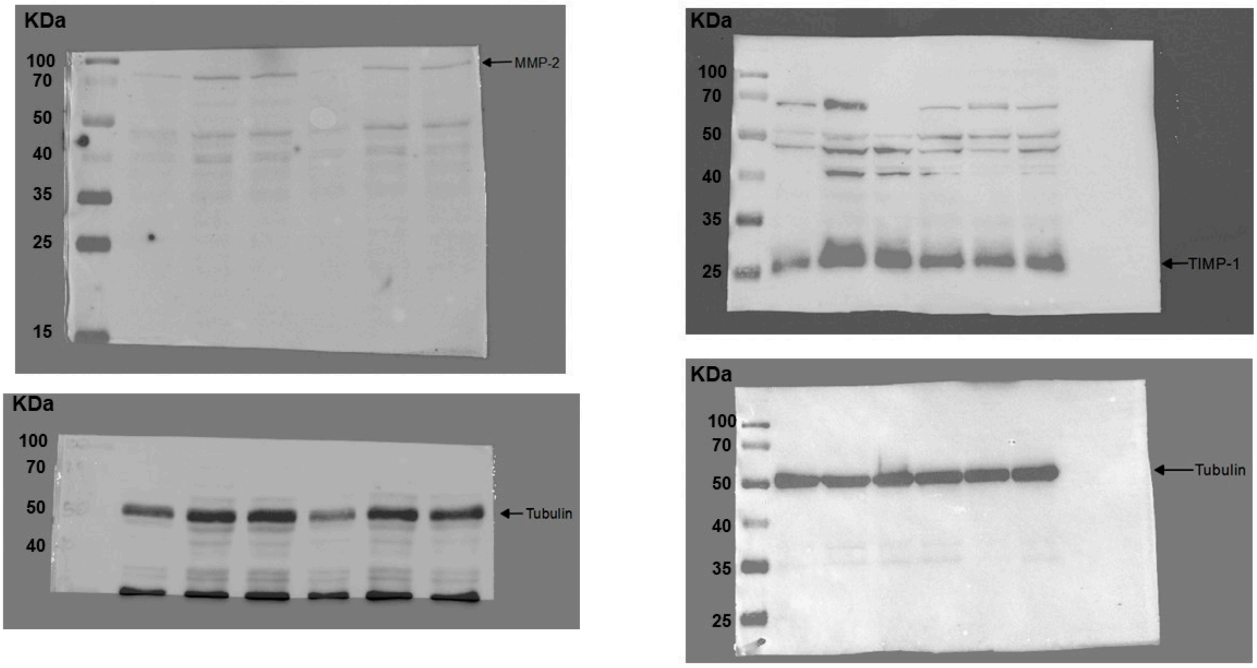


Figure 4

Detailed information about Figure 4.

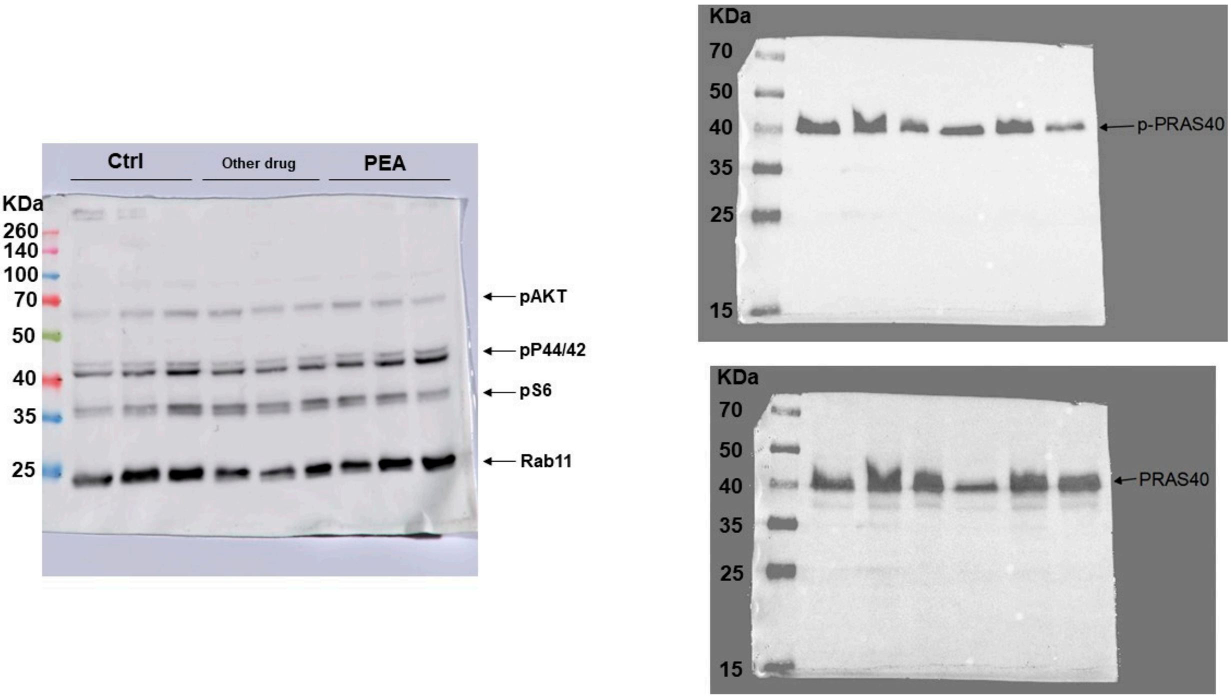


**Supplementary Figure 2**

Detailed information about Figure S2.

© 2021 by the authors. Licensee MDPI, Basel, Switzerland. This article is an open access article distributed under the terms and conditions of the Creative Commons Attribution (CC BY) license (http://creativecommons.org/licenses/by/4.0/).
